# MHC Class II Restricted Innate-Like Double Negative T Cells Contribute to Optimal Primary and Secondary Immunity to *Leishmania major*

**DOI:** 10.1371/journal.ppat.1004396

**Published:** 2014-09-18

**Authors:** Zhirong Mou, Dong Liu, Ifeoma Okwor, Ping Jia, Kanami Orihara, Jude Ezeh Uzonna

**Affiliations:** Department of Immunology, Faculty of Medicine, University of Manitoba, Winnipeg, Manitoba, Canada; Santa Casa Hospital Belo Horizonte, Brazil

## Abstract

Although it is generally believed that CD4^+^ T cells play important roles in anti-*Leishmania* immunity, some studies suggest that they may be dispensable, and that MHC II-restricted CD3^+^CD4^−^CD8^−^ (double negative, DN) T cells may be more important in regulating primary anti-*Leishmania* immunity. In addition, while there are reports of increased numbers of DN T cells in *Leishmania*-infected patients, dogs and mice, concrete evidence implicating these cells in secondary anti-*Leishmania* immunity has not yet been documented. Here, we report that DN T cells extensively proliferate and produce effector cytokines (IFN-γ, TNF and IL-17) and granzyme B (GrzB) in the draining lymph nodes and spleens of mice following primary and secondary *L. major* infections. DN T cells from healed mice display functional characteristics of protective anti-*Leishmania* memory-like cells: rapid and extensive proliferation and effector cytokines production following *L. major* challenge *in vitro* and *in vivo*. DN T cells express predominantly (> 95%) alpha-beta T cell receptor (αβ TCR), are *Leishmania*-specific, restricted mostly by MHC class II molecules and display transcriptional profile of innate-like genes. Using *in vivo* depletion and adoptive transfer studies, we show that DN T cells contribute to optimal primary and secondary anti-*Leishmania* immunity in mice. These results directly identify DN T cells as important players in effective and protective primary and secondary anti-*L. major* immunity in experimental cutaneous leishmaniasis.

## Author Summary

Although it is generally believed that CD4^+^ T cells mediate anti-*Leishmania* immunity, some studies suggest that CD3^+^CD4^−^CD8^−^ (double negative, DN) T cells may play a more important role in regulating primary anti-*Leishmania* immunity. Here, we report that DN T cells extensively proliferate and produce effector cytokines in mice following primary and secondary *L. major* infections. *Leishmania*-reactive DN T cells utilize αβ T cell receptor (TCR) and are restricted by MHC class II molecules. Strikingly, DN T cells from healed mice display functional characteristics of protective anti-*Leishmania* memory-like cells: rapid and extensive proliferation, effector cytokine production *in vitro* and *in vivo*, and accelerated parasite control following secondary *L. major* challenge. These results directly identify DN T cells as important players in protective primary and secondary anti-*L. major* immunity in experimental cutaneous leishmaniasis.

## Introduction

The spectrum of disease collectively called Leishmaniasis is caused by several species of protozoan parasites belonging to the genus *Leishmania*. The disease is currently endemic in 88 countries, affecting an estimated 12 million people with over 1.5–2 million new cases and 70,000 deaths each year [1]. Because *Leishmania* parasites reside mainly within macrophages, a strong cell-mediated immunity is required to control intracellular parasite replication and disease progression [2], [3], [4], [5], [6]. Experimental *L. major* infection in mice closely mimics the human cutaneous disease and is an excellent model for understanding the factors that regulate cell-mediated immunity. Resistance to cutaneous leishmaniasis is associated with strong IFN-γ response, which activates infected macrophages leading to nitric oxide and reactive oxygen species production and destruction of the intracellular parasites [4], [7], [8], [9].

Although it is generally believed that CD4^+^ T cells play a primary role in mediating anti-*Leishmania* immunity, a study suggests that they may be dispensable and that MHC II-restricted CD3^+^CD4^−^CD8^−^ (double negative, DN) T cells are critical for regulating primary anti-*Leishmania* immunity [10]. In addition, several studies have reported increased numbers of DN T cells in blood of *Leishmania*-infected patients [11], [12], dogs [13], and in spleens of *Leishmania*-infected mice [14]. These cells have been proposed to contribute to primary and vaccine-induced immunity against *Leishmania*. However, direct evidence implicating DN T cells in anti-*Leishmania* immunity has not yet been clearly documented. Here, we report for the first time, that infection with *L. major* leads to activation and proliferation of DN T cells in the draining lymph nodes (dLNs) and spleens of infected mice. These cells produce effector cytokines (IFN-γ and TNF), display functional characteristics of memory-like cells and contribute to optimal primary and secondary protection against *L. major* infection.

## Results

### DN T cells from healed mice proliferate and produce effector cytokines in response to *L. major-*infected BMDCs *in vitro*

**Figure 1:**
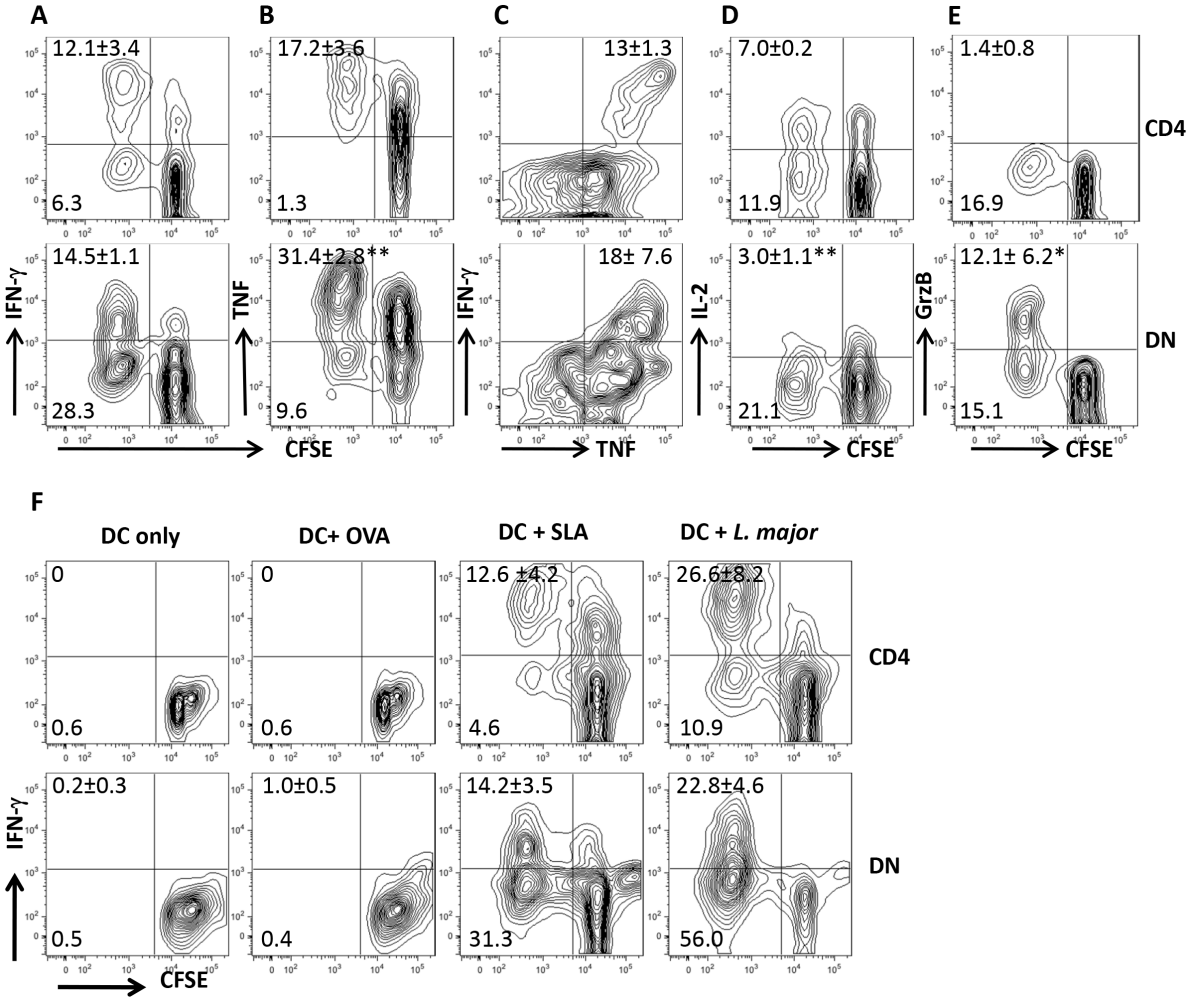
DN T cells from healed mice proliferate and produce effector cytokines following restimulation with *L. major*-infected BMDCs *in vitro*. Highly purified CFSE-labeled T cells from spleens of *L. major*-infected and healed C57BL/6 mice (> 12 weeks) depleted of CD8^+^ T cells (by treatment with anti-CD8 mAb) were co-cultured with *L. major*-infected BMDCs for 5 days and proliferation (A and B) and IFN-γ (A) and TNF (B) production by CD4^+^ and DN T cells were analyzed by flow cytometry following gating on CD3^+^ T cells. In addition, the co-production of IFN-γ and TNF by CD4^+^ and DN cells was also assessed (C). DN T cells do not produce appreciable amounts of IL-2 (D) but are strong producers of granzyme B (E). CFSE-labeled T cells were co-cultured with BMDCs only or pulsed with OVA (50 µg/ml), SLA (50 µg/ml), or freeze-thawed *L. major* (10^7^/ml) and proliferation and IFN-γ production by CD4^+^ and DN T cells were analyzed (F). Results are representative of 3 (A and B) and 2 (C–F) independent experiments (n = 3–4 mice) with similar results. *, p<0.05; **, p<0.01.

Recovery from natural or experimental *L. major* infection is associated with strong T cell proliferation and IFN-γ production in spleens and dLNs. To investigate the contribution of CD4^+^ T cells in this process, we co-cultured CD8^+^ T cell-depleted splenocytes from healed mice with *L. major*-infected BMDCs *in vitro*. Surprisingly, we found in addition to CD4^+^ T cells, strong proliferative and IFN-γ responses by CD3^+^CD4^−^CD8^−^ (DN) T cells (Fig. 1A and Fig. S1A and B). Proliferating DN T cells also produced TNF (Fig 1B), IL-17 (Fig. S2A) and little IL-2 (Fig. 1D), suggesting they are polyfunctional in cytokine production. Indeed, most of the IFN-γ-producing DN cells also co-produced TNF (Fig. 1C). Interestingly, although DN T cells proliferate significantly more than CD4^+^ T cells, their quantitative ability to produce IFN-γ and TNF was significantly lower than those of CD4^+^ T cells (Fig. S2B–D). In addition, DN T cells also produced GrzB (Fig. 1E), suggesting they may perform effector functions in *L. major*-infected mice. DN T cells from *L. major*-infected mice did not proliferate or produce IFN-γ following stimulation with OVA-loaded DCs (Fig. 1F), but were activated by DCs pulsed with SLA or freeze-thawed *L. major* (Fig. 1F). Collectively, these results suggest that the proliferation and cytokine production by DN T cells from healed mice are *L. major* specific.

### DN T cells proliferate and produce IFN-γ *in vivo* after rechallenge with *L. major* and display memory markers

**Figure 2:**
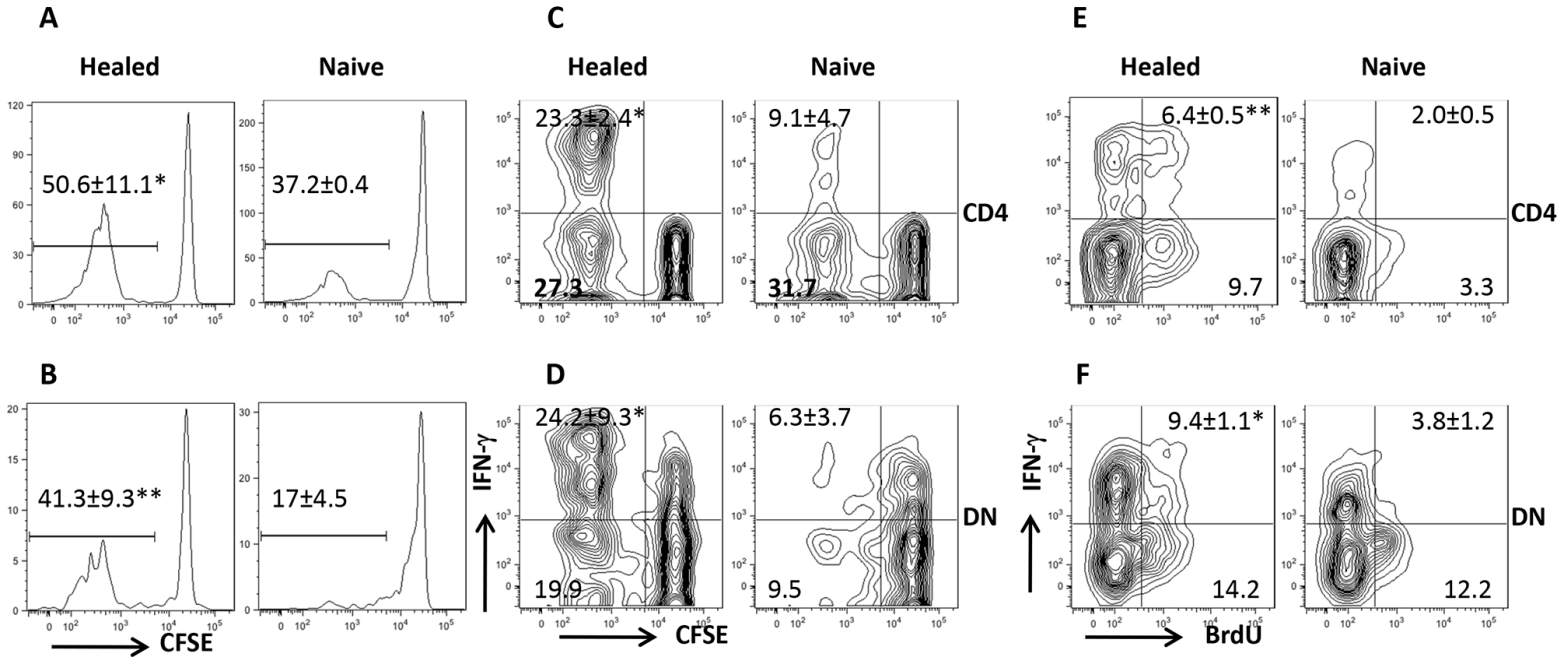
DN T cells proliferate and produce IFN-γ *in vivo*. Highly enriched (> 98%) CFSE-labeled CD90.2^+^ T cells from naïve or healed Thy1.2 mice were adoptively transferred into naïve Thy1.1 recipients that were challenged with 5×10^6^ *L. major* the next day. Seven days after challenge, mice were sacrificed and cell proliferation (A, B) and IFN-γ production (C, D) by CD4^+^ (A, C) and DN (B, D) T cells were analyzed directly *ex vivo* by gating on Thy1.2^+^CD3^+^ donor cell population. For BrdU incorporation assay, naïve or healed mice were challenged with *L. major*, treated daily with BrdU in drinking water and by i.p. injection, sacrificed after 7 days and BrdU incorporation and IFN-γ expression by CD4^+^ (E) and DN (F) T cells were analyzed. Results are presented as means +/− SE and representative of 2 independent experiments (n = 3–4 mice) with similar results. *, p<0.05; **, p<0.01 vs. naïve controls.

**Figure 3:**
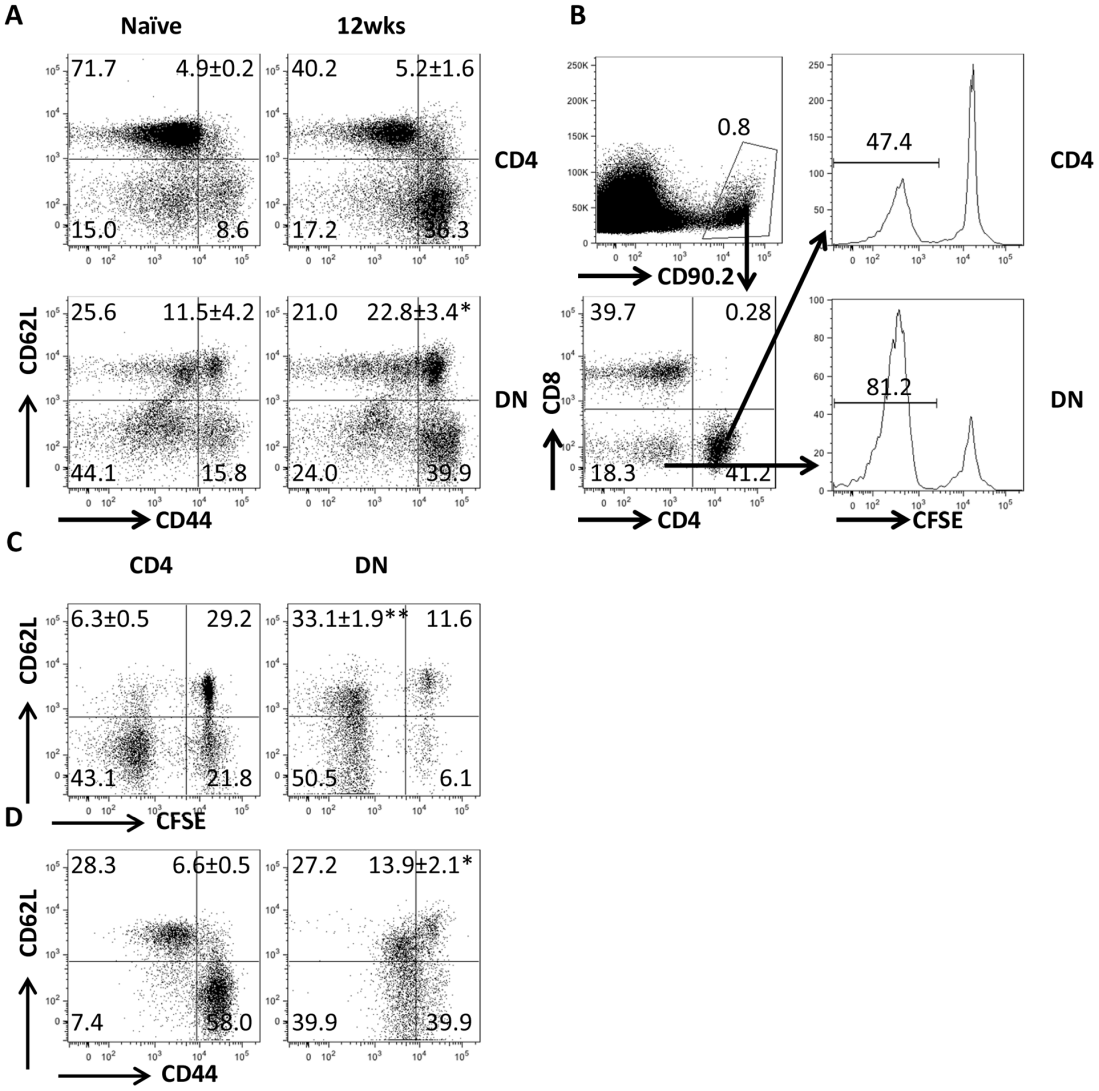
DN T cells from *L. major*-infected mice display phenotypic characteristic of memory cells. (A) Direct *ex vivo* expression of CD62L and CD44 on DN and CD4^+^ T cells from dLNs of naive and healed mice. (B–D) Phenotypic characteristics of *Leishmania*-reactive DN T cells *in vivo*. CFSE-labeled splenocytes from healed CD90.2 mice were transferred into naïve CD90.1 recipients that were then challenged with 5× 10^6^ *L. major*. Challenged mice were sacrificed after 7 days and proliferation of donor CD4^+^ and DN T cells in dLNs was assessed directly *ex vivo* (B). In addition, the expression of CD62L within proliferating (C) and CD44^hi^ (D) donor CD4^+^ and DN T cells was also assessed. Results are representative of 2 independent experiments with similar results using pooled cells from 3 mice with similar results. *, p<0.05; **, p<0.01; vs. CD4^+^ T cells.

Our co-culture system showed that *Leishmania*-specific DN T cells are activated following *in vitro* recall response. To determine whether DN T cells are activated *in vivo*, we adoptively transferred CFSE-labeled T cells from healed Thy1.2 mice into naive Thy1.1 mice that were then challenged with *L. major* the next day. Both CD4^+^ and DN T cells from healed donor mice showed extensive proliferation and IFN-γ production compared to those from naive mice (Fig. 2A–D). The *in vivo* relevance of DN T cell response was further confirmed by BrdU incorporation (Fig. 2E and F). Interestingly and similar to CD4^+^ T cells, the percentage of proliferating and IFN-γ-producing DN T cells in healed mice were significantly higher than those in naïve mice following *L. major* challenge, suggesting that DN T cells display functional characteristics of memory T cells (rapid proliferation and cytokine production). Indeed, we found that the percentage of DN T cells in lymph nodes (Fig 3A) of healed mice that express CD62L^hi^CD44^hi^ (central memory-like) was significantly higher (p<0.05) than those in naive mice (Fig. 3B). Following adoptive transfer of whole T cells from healed mice and subsequent *L. major* challenge, almost all the proliferating donor CD4^+^ T cells downregulated their CD62L expression (i.e. were CD62L^lo^). In contrast, the proliferating DN T cells contained an almost equal proportion of CD62L^lo^ and CD62L^hi^ populations (Fig. 3C). In addition, more DN T cells were CD62L^hi^CD44^hi^ compared to CD4^+^ T cells (Fig. 3D).

### DN T cells predominately express αβ-TCR and do not have regulatory properties

**Figure 4:**
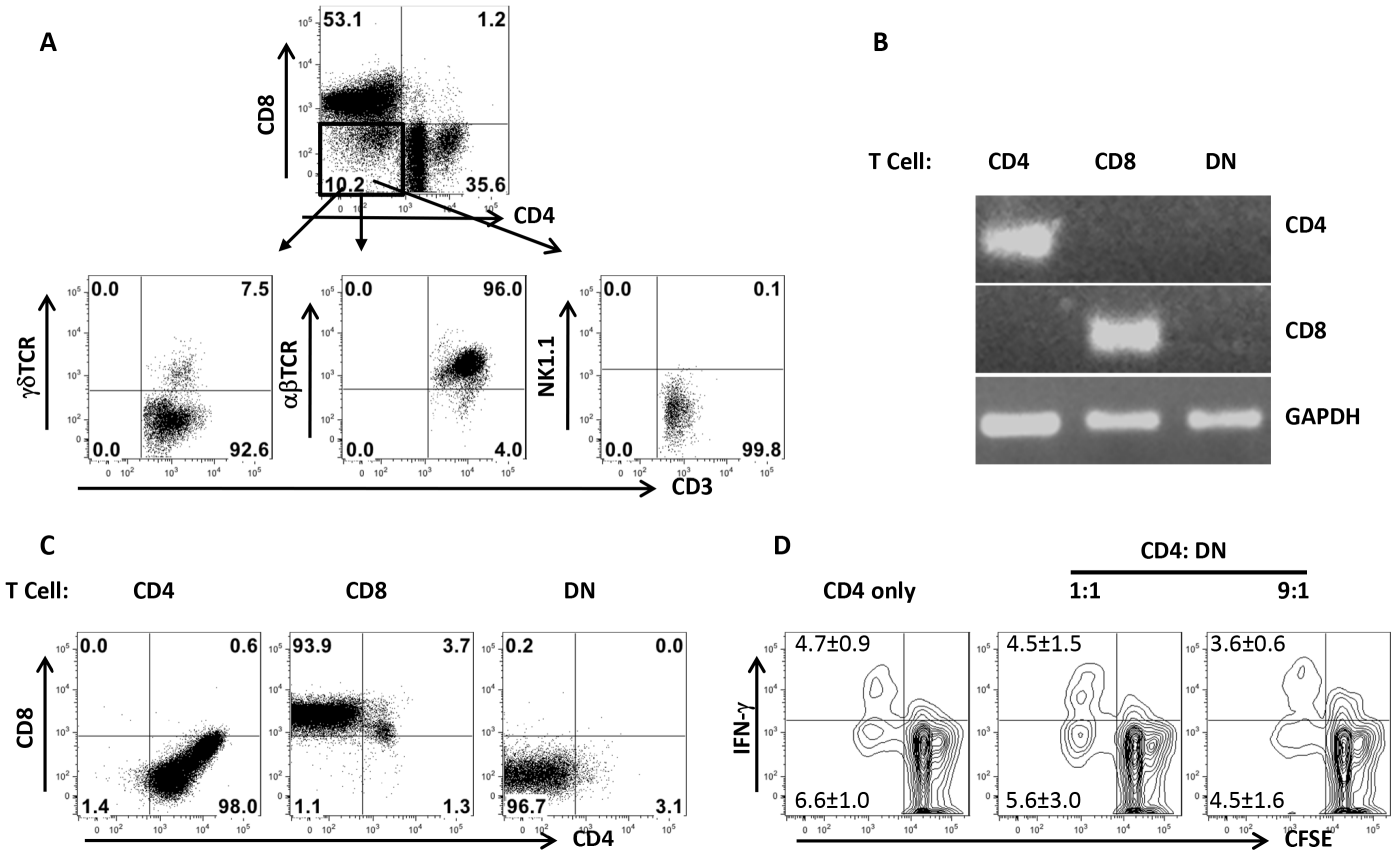
DN T cells predominately express αβ-TCR and do not have regulatory properties. Highly purified T cells from healed mice were co-cultured with *L. major*-infected BMDCs for 5 days and the expression of αβ, γδ and NK1.1 molecules by DN T cells was analyzed by flow cytometry (A). In some experiments, CD4^+^, CD8^+^ and DN T cells were enriched by cell sorting and the expression of CD4 and CD8 gene transcripts in the enriched cells was assessed by RT-PCR (B). Highly purified CD4^+^, CD8^+^ and DN T cells were further stimulated with *L. major*-infected BMDCs for 5 days and CD4 and CD8 expression was analyzed by flow cytometry (C). Purified CFSE-labeled CD4^+^ cells from healed mice were cultured *L. major* infected BMDCs either alone or with DN T cells for 5 days and CD4^+^ T cell proliferation and IFN-γ production were analyzed by flow cytometry (D). Results are representative of 2–3 independent experiments with similar results.

In addition to αβ T cells, NKT and γδ T cells also do not express CD4 and CD8 molecules. To determine whether *Leishmania*-reactive DN T cells are NKT and γδ T cells, we assessed the expression of αβ, γδ and NK1.1 molecules on DN T cells by flow cytometry. As shown in Fig. 4A, DN T cells predominately (> 90%) expressed αβ TCR and not NK1.1 and γδ molecules, indicating that they are not NKT or γδ T cells. To further determine whether DN T cells are CD4^+^ or CD8^+^ T cells that have down-regulated their surface molecules following activation, we assessed highly enriched (> 99% purity, Fig. S3) DN, CD4^+^ and CD8^+^ T cells for CD4 and CD8 transcripts by RT-PCR. DN T cells did not express CD4 and CD8 mRNA (Fig. 2B), suggesting they are not CD4^+^ or CD8^+^ T cells that have down-regulated their surface molecules. In addition, highly purified CD4^+^, CD8^+^ and DN T cells maintained their respective phenotypes following *in vitro* restimulation for 5 days with *L. major*-infected BMDCs (Fig. 4C). To determine whether *Leishmania*-reactive DN T cells display regulatory properties as previously reported in other systems [12], [15], [16], we co-cultured CD4^+^ and DN T cells with *L. major*-infected BMDCs and assessed CD4^+^ T cell proliferation and IFN-γ production by flow cytometry. DN T cells did not affect CD4^+^ cell proliferation and IFN-γ production (Fig. 4D), suggesting that they do not exhibit regulatory/suppressive properties.

### DN T cells are mostly restricted by MHC II

**Figure 5:**
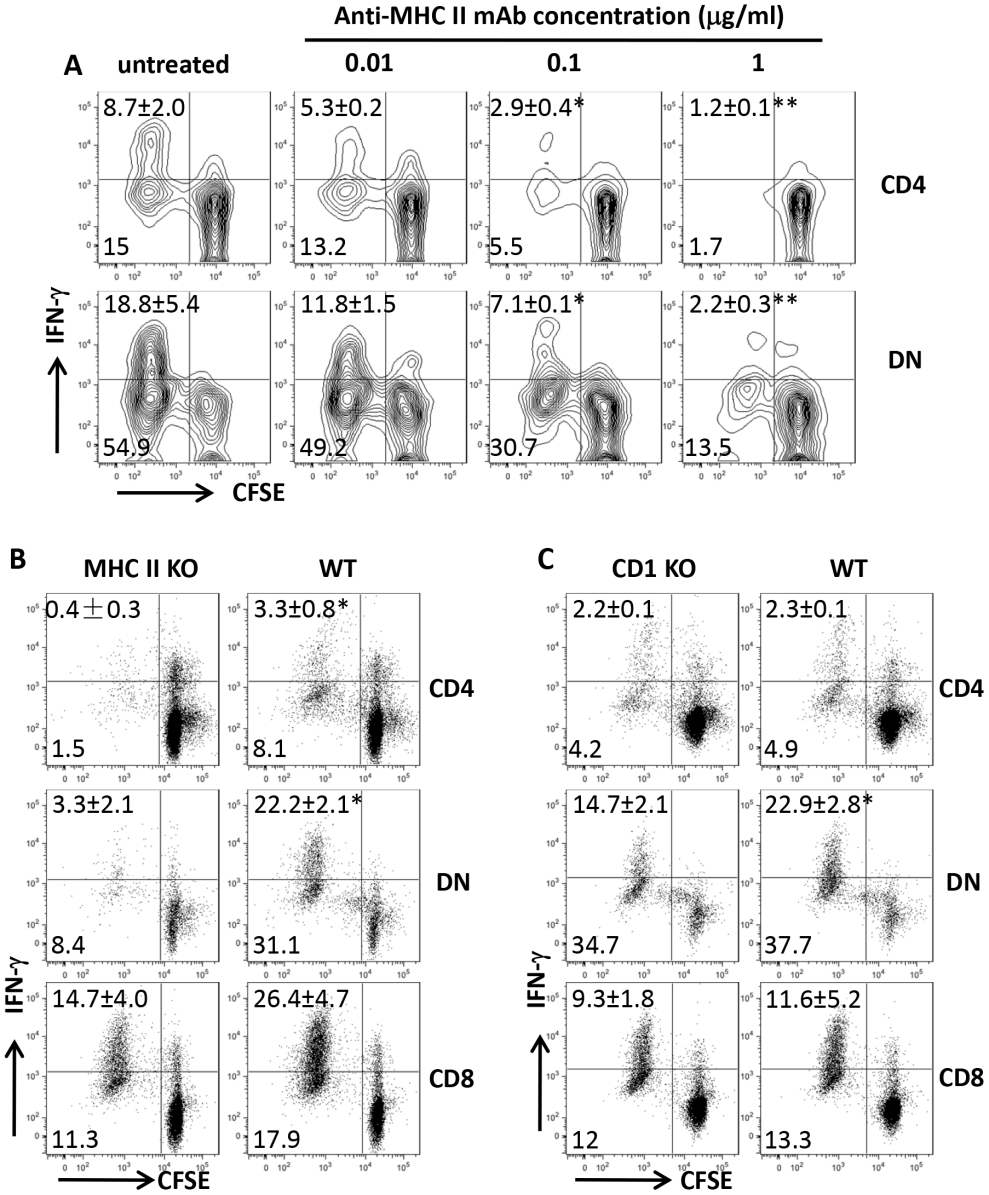
DN T cells are restricted by MHC II molecules. CFSE-labeled highly purified T cells from spleens of healed mice were co-cultured with *L. major*-infected BMDCs with or without varying concentrations of anti-MHC II mAb and proliferation and IFN-γ production by CD4^+^ and DN T cells were assessed after 5 days (A). In some experiments, the ability of infected BMDCs from MHC II KO (B) or CD1d KO (C) mice to induce proliferation and IFN-γ production by CD4^+^, CD8^+^ and DN T cells was compared with those from WT controls. Results are representative of 2–3 independent experiments with similar results. *, p<0.05; **, p<0.01; mAb treated vs. isotype controls or KO vs. WT groups.

To determine whether *Leishmania*-reactive DN T cells are restricted by MHC II molecule, we co-cultured highly enriched T cells from healed mice with infected BMDCs in the presence or absence of anti-MHC II antibodies. Anti-MHC II antibodies blocked proliferation and IFN-γ production by both CD4^+^ and DN T cells in a dose-dependent manner (Fig. 5A). In addition, *L. major*-infected BMDCs from MHC II KO mice failed to induce proliferation and IFN-γ production by DN and CD4^+^ T cells (Fig. 5B). In contrast, proliferation and IFN-γ production by DN T cells were minimally affected following co-culture with infected BMDCs from CD1d KO mice (Fig. 5C), confirming that DN T cells are mostly restricted by MHC II molecules.

### DN T cells are activated during primary *L. major* infection

**Figure 6:**
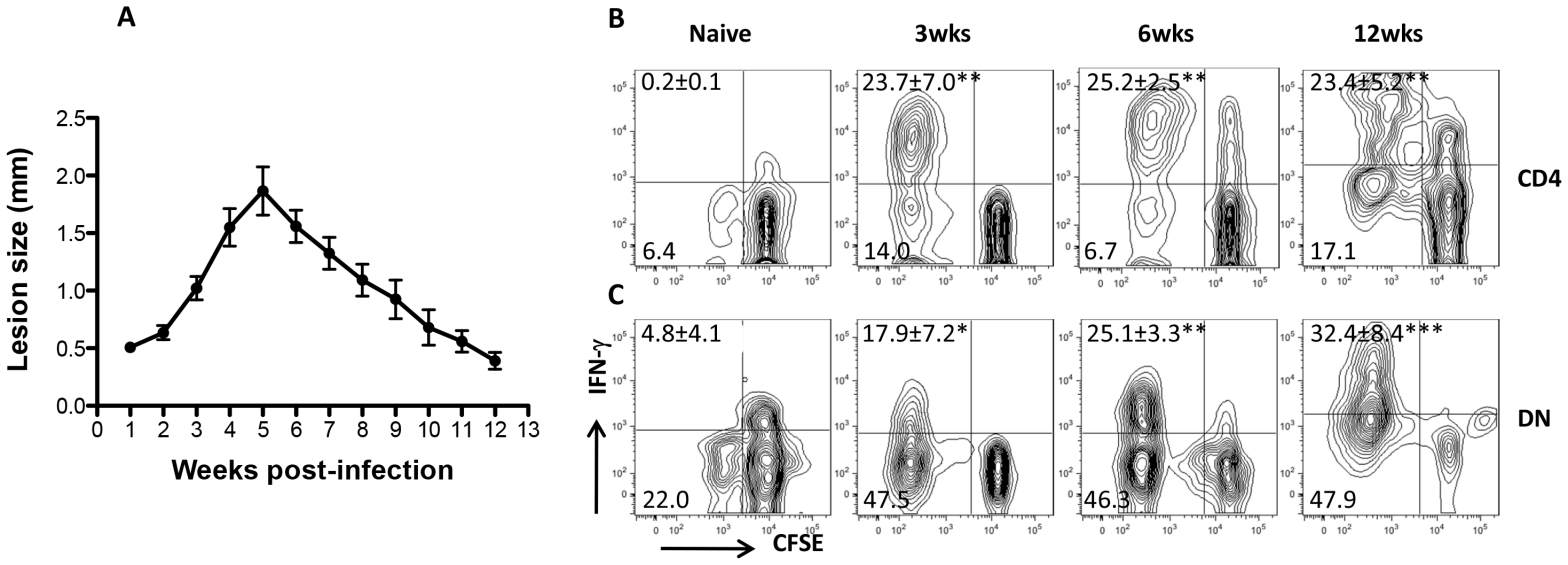
DN T cells are activated during primary *L. major* infection. C57BL/6 mice were infected with 2×10^6^ *L. major* and lesion progression was monitored weekly with calipers (A). At indicated times, infected mice were sacrificed and pooled splenic and dLN T cells were co-cultured with *L. major*-infected BMDCs for 5 days and cell proliferation and IFN-γ production by CD4^+^ (B) and DN (C) T cells were analyzed by flow cytometry. Results are representative of 2–3 independent experiments (n = 3–4 mice) with similar results. *, p<0.05; **, p<0.01; ***, p<0.001 infected vs. naïve (uninfected) controls.

We found that *Leishmania*-reactive DN T cells are recalled in healed mice following *L. major* challenge *in vitro* and *in vivo* suggesting that they may be induced following primary infection. To determine this, we assessed CD4^+^ and DN T cells response in the dLNs and spleens of infected mice C57BL/6 mice at different times after infection corresponding to early, peak and resolution of lesion progression (Fig. 6A). As expected, there was strong CD4^+^ T cell response (proliferation and IFN-γ production, Fig. 6B) at all times (3, 6 and 12 weeks) post-infection. Similarly, DN T cells from infected mice also strongly proliferated and produced IFN-γ following restimulation with infected BMDCs (Fig. 6C). In contrast, CD4^+^ and DN T cells from naïve mice did not proliferate or produce IFN-γ upon stimulation with *L. major*-infected BMDCs (Fig. 6B and C). Collectively, these results show that *Leishmania*-reactive DN T cells are induced during primary *L. major* infection and could contribute to anti-*Leishmania* immunity.

### DN T cells contribute to optimal primary and secondary immunity against *L. major*

**Figure 7:**
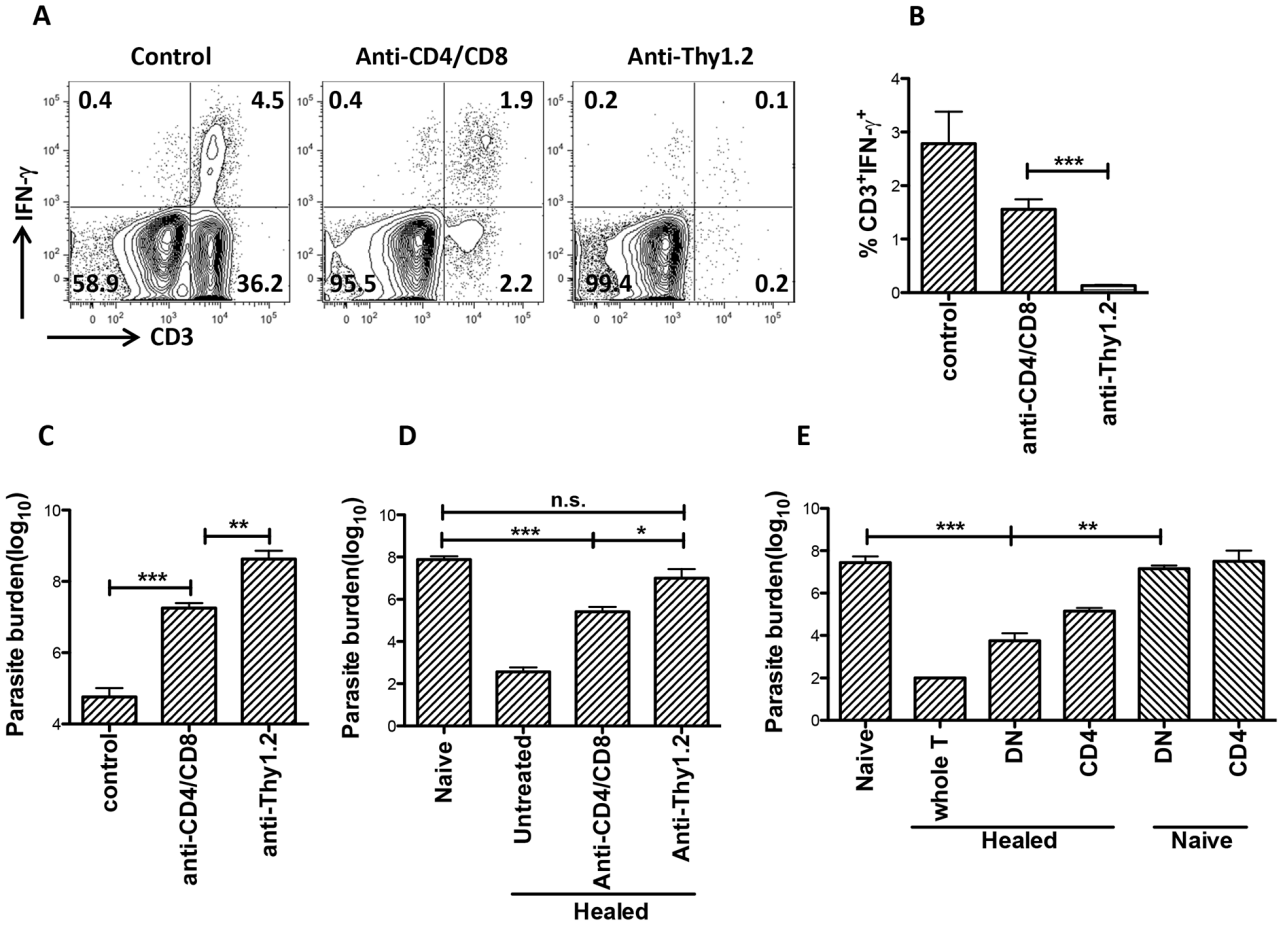
DN T cells contribute to optimal primary and secondary immunity against *L. major* infection. Naïve (A–C) or healed (> 12 weeks post infection, D) C57BL/6 mice were treated with anti-Thy1.2 or combined anti-CD4/anti-CD8 mAbs once weekly and challenged with 5×10^6^ *L. major* 3 days after the first antibody treatment. At 3 weeks post-challenge, mice were sacrificed and dLN cells were stained directly *ex vivo* the percentage of CD3^+^IFN-γ^γ^ cells was determined by flow cytometry (A and B). Parasite burden in the challenged footpads was determined by limiting dilution (C and D). Highly enriched CD4^+^ and DN (> 99% purity) T cells from healed or naïve mice were transferred into naïve mice that were then challenged with 5×10^6^ *L. major*. Challenged mice were sacrificed after 3 weeks to determine parasite burden (E). Results are representative of 2 independent experiments (n = 3–4 mice) with similar results. *, p<0.05; **, p<0.01; ***, p<0.001; anti-CD4/CD8 vs. anti-Thy1.2 mAb groups and healed DN vs. naïve DN recipient groups (E).

To determine if DN T cells contribute to primary immunity against *L. major*, we selectively depleted CD4^+^ and CD8^+^ or all T cells by treatment with anti-CD4/CD8 or anti-Thy1.2 mAbs, respectively, during the course of primary *L. major* infection (Fig. S4). Mice depleted of both CD4^+^ and CD8^+^ T cells still had some IFN-γ-producing CD3^+^ DN T cells (Fig. 7A and 7B) and harbor significantly (p<0.01) lower parasite burden (Fig. 7C) compared to those depleted of all T cells (by anti-Thy1.2 mAb treatment), indicating that DN T cells contribute to optimal control of parasite proliferation during primary *L. major* infection.

Next, we used both *in vitro* and *in vivo* approaches to investigate whether DN T cells contribute to secondary anti-*Leishmania* immunity. Highly purified DN T cells from healed (but not naïve) mice significantly (p<0.05) inhibited parasite proliferation in infected BMDMs and this effect was comparable to those of CD4^+^ T cells (Fig. S5A and B). These results provide direct *in vitro* evidence that DN T cells could control parasite growth in *L. major*-infected BMDMs.

Next, we used two different experimental approaches to determine whether DN T cells contribute to secondary anti-*Leishmania* immunity *in vivo*. First, we selectively depleted CD4^+^ and CD8^+^ or all CD3^+^ T cells (as in Fig. 7A above) in healed mice and after 24 hr, rechallenged them with *L. major*. As shown in Fig. 7D, CD4^+^ and CD8^+^ T cells-depleted mice, which still had DN T cells, retained some level of infection-induced resistance as evidenced by significantly (p<0.01) lower parasite burden compared to naïve mice (primary infection). In contrast, depletion of all CD3^+^ T cells completely abrogated secondary immunity (Fig. 7D). Second, we assessed the ability of highly enriched (purity > 96%, Fig. S6) DN T cells from healed mice to protect naïve animals against virulent *L. major* challenge. Adoptively transferred DN T cells from healed mice protected naïve mice against virulent *L. major* challenge as evidenced by significantly lower parasite burden (Fig. 7E). Collectively, these *in vitro* and *in vivo* observations strongly implicate *Leishmania*-reactive DN T cells in contributing to optimal anti-*Leishmania* immunity in mice.

### Increased transcriptional activity of innate genes in *Leishmania*-reactive DN T cells

**Figure 8:**
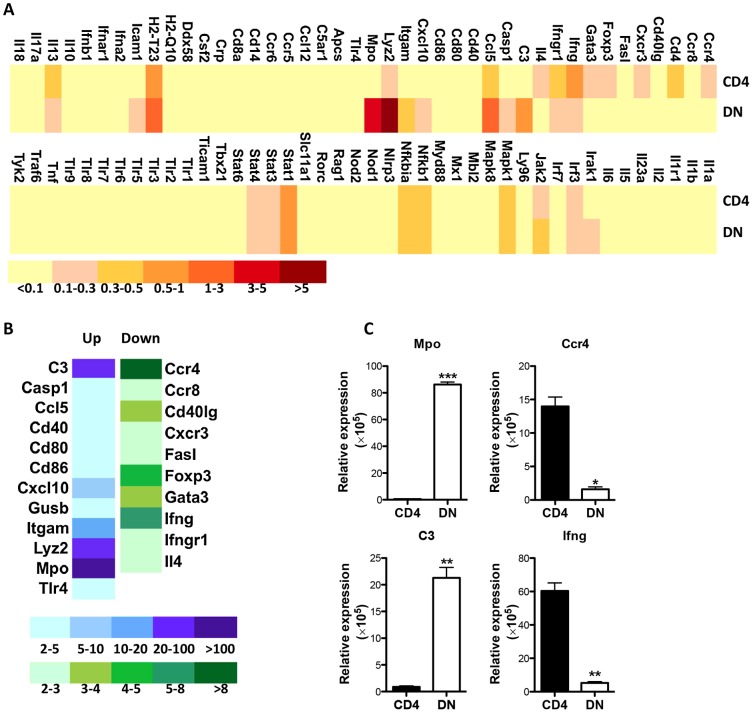
Enhanced transcription of innate genes in *Leishmania*-reactive DN T cells. CFSE-labeled pooled splenocytes and dLN cells from healed mice were stimulated with *L. major*-infected BMDCs for 5 days and total mRNA was isolated from CFSE^lo^ (proliferating) CD4^+^ and DN T cells that were purified by cell sorting and used for PCR array. (A) Heat map showing gene expression profile by proliferating CD4^+^ and DN T cells. Relative expression levels were shown following normalization with internal controls. The genes expressing greater than two fold changes between CD4^+^ and DN T cells were further analyzed (B) and their expression levels were validated by qPCR following normalization with the housekeeping gene, 18S (C). *, p<0.05; **, p<0.01; ***, p<0.001.

Apart from lacking CD4 molecules, DN T cells display functional characteristics similar to CD4^+^ T cells (MHC-II restriction, proliferation, IFN-γ production and parasite control). To further investigate how *Leishmania*-reactive DN T cells differ from CD4^+^ T cells, we compared the transcriptional profile of proliferating DN and CD4^+^ T cells following restimulation with *L. major*-infected BMDCs. Although most of the 84 mouse innate and adaptive immune genes showed similar pattern and level of expression in both cell types, some genes were preferentially upregulated or downregulated in DN T cells compared to CD4^+^ T cells (Fig. 8A). The gene transcripts showing ≥ 2 folds difference in DN T cells were further analyzed and validated by quantitatively real-time PCR (Fig 8B and C). Interestingly, most of the upregulated transcripts in DN T cells were genes associated with innate immune responses, including C3, Mac-1 (CD11b), myeloperoxidase (Mpo), lysozyme, etc. In contrast, the downregulated transcripts (relative to CD4^+^ T cells) included genes associated with adaptive immunity, including CCR4, Foxp3, Gata-3, etc. Collectively, these results suggest that despite mediating anti-*Leishmania* immunity (akin to CD4^+^ T cells), *Leishmania*-reactive DN T cells are phenotypically distinct from conventional CD4^+^ T cells.

## Discussion

We show here that DN T cells proliferate and produce effector cytokines in secondary lymphoid organs of mice following primary and secondary *L. major* challenges. DN T cells from healed mice display functional characteristics of anti-*Leishmania* memory-like cells: they rapidly proliferate and produce effector cytokines (TNF and IFN-γ) in response to *L. major* challenge *in vitro* and *in vivo* and mediate infection-induced immunity (rapid protection) following adoptive transfer *in vivo*. *Leishmania*-reactive DN T cells express predominantly αβ TCR, are restricted by MHC class II molecules, lack immunoregulatory properties and display transcriptional profile distinct from conventional CD4^+^ T cells. To the best of our knowledge, this is the first extensive characterization and demonstration of the protective ability of *Leishmania*-reactive DN T cells *in vitro* and *in vivo*.

It is generally believed that CD4^+^ T cells play a dominant role in anti-*Leishmania* immunity. However, the finding that CD4 deficient mice were resistant while MHC class II deficient mice were highly susceptible to *L. major* challenged this dogma [10] and suggests that MHC II-restricted CD4^−^CD8^−^ T cells may be more important in regulating primary anti-*Leishmania* immunity. Indeed, several studies have reported the expansion of CD3^+^CD4^−^CD8^−^ (DN) T cells in the blood of *Leishmania*-infected patients and dogs, and in spleens of *Leishmania*-infected mice [11], [12], [13], [14]. These cells have been proposed to contribute to primary and vaccine-induced immunity although a concrete evidence implicating them in immunity has not yet been demonstrated. Our studies directly show the importance of *Leishmania*-reactive DN T cells in mediating optimal primary and secondary anti-*Leishmania* immunity in mice.

The precise origin and development of peripheral DN T cells is not clearly understood and is controversial. Some reports suggest that DN T cells originate in the thymus by escaping negative selection [17], [18], [19]. In contrast, several reports suggest that DN T cells are generated in the periphery rather than in the thymus [19], [20], [21], [22]. These cells comprise about 1–5% of total T cells in non-transgenic mice and in humans [11], [23] making them difficult to isolate and subsequently study. TCR transgenic [24] or lpr (Fas mutation) mice [19], [25], which present increasing accumulation of DN T cells are widely used to investigate the function and developmental origin of DN T cells. DN T cells have been shown to influence long-term allograft survival [24], [26], [27], prevent the development of autoimmune disease [28], [29], [30], and contribute to control of intracellular pathogens [31], [32]. In addition, DN T cells have been shown to possess immunoregulatory and alloreactive properties, inhibit autoreactive CD4^+^ T cells and mediate MHC I-restricted killing of allogenic target cells [20], [24], [25]. Our studies show that *Leishmania*-reactive DN T cells are restricted by MHC class II and may not have immunoregulatory properties because they failed to suppress CD4^+^ T cell proliferation *in vitro* (Fig. 4D). Rather, a large percentage of proliferating DN T cells produced IFN-γ, TNF, IL-17 and GrzB, which is consistent with their effector functions as seen in other studies [11], [12], [29].

Previous studies that have reported the expansion and possible protective role of DN T cells in leishmaniasis focused mainly on primary *Leishmania* infection [11], [12], [13], [14]. We extend these studies during secondary immunity by showing rapid expansion and effector functions (cytokine production and parasite control) by DN T cells following challenge infection. Healed mice had more proliferating and IFN-γ-producing DN T cells compared with naive mice following *L. major* challenge (Fig. 2), and adoptive transfer of DN T cells from healed (but not naïve mice) rapidly protected naïve mice against virulent *L. major* change. Moreover, DN T cells from healed mice expressed high levels of CD44 and majority of them were CD62L^hi^CD44^hi^, which are characteristics markers expressed by central memory-like cells. Collectively, these results suggest that DN T cells display functional characteristics of memory cells and contribute to optimal secondary immunity against *L. major*.

How do DN T cells mediate their anti-*Leishmania* immunity? We speculate that this may be related in part to their ability to produce IFN-γ and TNF, key cytokines that activate infected macrophages leading to intracellular parasite killing. Indeed, we found that *Leishmania*-reactive DN T cells in the spleens and lymph nodes are highly proliferative and produce IFN-γ, TNF and granzyme B. Importantly, we also found that DN T cells from immune mice were recruited to and proliferate at the infected footpads (Fig. S7). In addition, our *in vitro* co-culture experiments with infected BMDMs and highly enriched DN T cells show that suppression of parasite proliferation was associated with increased nitric oxide production, a key effector molecule that mediate destruction of parasites in infected cells.

The findings that DN T cells mediate comparable (or even superior) protection against *L. major in vitro* and *in vivo* may challenge the dogma that CD4^+^ T cells are the major T cell subset that mediates anti-*Leishmania* immunity. Indeed, the proliferation of DN T cells was either comparable or sometimes higher than those of CD4^+^ T cells following *in vitro* or *in vivo L. major* challenge (see Figs. 1–3). Interestingly, although the percentage of IFN-γ-producing DN T cells was sometimes higher than those of CD4^+^ T cells, their MFI was significantly lower (Fig. 1C), an observation that explain the relatively lower IFN-γ transcripts in DN compared to CD4^+^ T cells (Fig. 8). In addition, the numbers of *Leishmania*-reactive CD4^+^ T cells were quantitatively (∼ 3–4 fold) higher than those of DN T cells. Thus, despite their superior proliferative response, DN T cells may still play a subordinate role to CD4^+^ T cells *in vivo*. Furthermore, it is conceivable that CD4^+^ T cells may be required for proper activation and effector functions of DN T cells. In line with this, we have observed that proliferation and IFN-γ production by highly enriched DN T cells is impaired in cultures devoid of immune CD4^+^ T cells *in vitro* and *in vivo* (Fig. S8). It is conceivable that DN T cells may assume increased roles in the absence of CD4^+^ T cells. For example, SIV infection in nonhuman primates does not result in immune dysfunction and progression to simian AIDS because DN T cells partially compensate for defective CD4^+^ T cell functions upon SIV-induced CD4^+^ T cell depletion in these animals [33], [34]. Similarly, a strong DN T cell-mediated HIV Gag-specific response has been associated with seronegativity in HIV-exposed individuals [35].

It is interesting that the expression of genes associated with innate immune responses including C3, were significantly higher in *Leishmania*-reactive DN T cells than in CD4^+^ T cells. While commonly associated with initiation of inflammation and critical molecule involved in first line of defense against pathogens, the complement proteins, particularly C3 and its degradation fragments are also known to prominently influence the adaptive immunity [36], [37]. Recent studies have been shown that some subset of T cells express C3 and that its intracellular activation is not only required for homeostatic T cell survival [38], but also in optimal Th1 induction and differentiation into effector cytokine (particularly IFN-γ) production [38], [39]. It is conceivable that C3-expressing DN T cells in *L. major*-infected mice might be involved in IFN-γ production leading to effective macrophage activation, nitric oxide production and parasite killing.

Collectively, our studies provide direct evidence for DN T cells in mediating anti-*Leishmania* immunity akin to CD4^+^ T cells. We propose that DN T cells complement CD4^+^ T cells to mediate efficient primary and secondary anti-*Leishmania* immunity in mice. In the absence of DN T cells, the induction of effective anti-*Leishmania* immunity may be either delayed or impaired. In a recent preliminary study, we observed impaired induction of DN T cells in spleens and draining lymph nodes of *L. major*-infected highly susceptible BALB/c mice. It would be interesting to determine whether the susceptibility of BALB/c mice to *L. major* infection is related in part to this impaired expansion of DN T cells. Collectively, our studies clearly identify DN T cells as important subset of T cells that contribute to optimal anti-*Leishmania* immunity.

## Materials and Methods

### Ethics statement

All mice were kept at the University of Manitoba Central Animal Care Services (CACS) facility in accordance to the Canadian Council for Animal Care guidelines. The University of Manitoba Animal Use Ethics Committee approved all studies involving animals, including infection, humane endpoints, euthanasia and collection of samples.

### Mice

Six to 8 wk-old female C57BL/6 (Thy1.2) mice were obtained from Charles River, St Constante PQ, Canada. Thy1.1 and MHC class II deficient (MHC II KO) C57BL/6 mice were purchased from The Jackson Laboratory (Bar Harbor, ME). Female CD1d deficient C57BL/6 mice were kindly supplied by Dr. Xi Yang from a breeding colony maintained at the University of Manitoba Central Animal Care Services (CACS) Facility.

### Infection protocol and parasite quantification

*Leishmania major* parasites (MHOM/80/Fredlin) were grown in M199 culture medium (Sigma, St. Louis, MO) supplemented with 20% heat inactivated FBS (HyClone, Logan, UT), 2 mM glutamine, 100 U/ml penicillin, and 100 µg/ml streptomycin. For infection, mice were injected with 2×10^6^ (primary infection) or 5×10^6^ (secondary infection) stationary-phase promastigotes in 50 µl PBS suspension into the right (primary) or left (secondary) hind footpad. Lesion sizes were monitored weekly by measuring footpad swelling with calipers. Parasite burden in the infected footpads was determined by limiting dilution assay. Parasite titers were determined from the highest dilution at which growth was visible.

### Bone marrow-derived macrophages (BMDMs) and dendritic cells (BMDCs) and *in vitro* infection

Bone marrow cells were isolated from the femur and tibia of naïve C57BL/c mice and differentiated into macrophages using complete medium supplemented with 30% L929 cell culture supernatant as previously described [40]. BMDCs were differentiated in petri dishes in the presence of rmGM-CSF (20 ng/ml; Peprotech, Rocky Hill, NJ). BMDMs and BMDCs were infected at a cell-to-parasite ratio of 1∶5 and after 6 hr, free parasites were washed away and infected BMDCs were used to stimulate purified CD3^+^, CD4^+^ or DN T cells from naïve or healed mice *in vitro*. To assess the ability of CD4^+^ or DN T cells to control parasite proliferation, infected BMDMs were co-cultured with CD4^+^ or DN T cells and parasite proliferation in infected BMDMs was determined at different times by counting Giemsa-stained cytospin preparations under light microscope at ×100 (oil immersion) objective.

### *In vitro* recall responses

Infected mice were sacrificed and spleens and dLNs were collected and made into single-cell suspensions. Cells were labeled with CFSE dye (1.5 mM; Molecular Probes, Eugene, OR) and resuspended at a concentration of 2×10^6^ cells per milliliter in RPMI 1640 supplemented with 10% heat-inactivated FBS, 100 U/ml penicillin, 100 µg/ml streptomycin, and 5×10^−5^ M 2-mercaptoethanol (complete medium), plated with 100 µl per well in 96-well tissue culture plates, and stimulated with infected BMDCs (BMDC: T cell = 1∶100) or soluble anti-CD3/CD28 mAb (1 µg/ml; BioLegend, San Diego, CA). After 5 days, proliferation and cytokine production were determined by flow cytometry. In some experiments, CFSE-labeled T cells from spleens and dLNs of infected mice were co-cultured with *L. major*-infected WT, MHC II KO, or CD1d KO BMDCs for 5 days, stimulated with PMA, BFA and ionomycin for 4–6 hr and proliferation, IFN-γ, TNF, IL-2, IL-17 and GrzB expression by different T cell subsets were analyzed by flow cytometry. In some experiments, anti-MHC II antibodies were used to block MHC II-TCR interaction *in vitro*.

### Purification of T cell subsets, cell labeling and adoptive transfer experiments

Healed (> 12 weeks post-infection) Thy1.2 C57BL/6 mice were sacrificed and single-cell suspensions from the dLNs and spleens were made. T cells (Thy1.2^+^) were enriched by positive selection using mouse CD90.2 (Thy1.2) selection kit according to the manufacturer's protocols (StemCell Technologies, Vancouver, BC). Enriched T cells (> 98% purity) were labeled with CFSE dye, and 10^7^ cells were adoptively transferred into naive congenic (Thy1.1) mice by tail vein injection. After 24 hr, the recipient mice were challenged with 5×10^6^ *L. major*, sacrificed after 7 days and cell proliferation and IFN-γ expression by donor (Thy1.2) cells in the dLNs and spleens were determined directly *ex vivo*.

### Enrichment of DN T cells

For *in vitro* co-culture experiments, DN (CD3^+^CD4^−^CD8^−^) and CD4^+^ T cells were purified from pooled spleens and dLNs of healed or naïve mice by cell sorting (FACSAria III, BD Biosciences). For *in vivo* adoptive transfer studies, DN T cells were enriched using a combination of *in vivo* depletion and positive selection. Briefly, *L. major*-infected and healed mice (> 12 weeks post-infection) were first injected with 200 µl GK1.5 and TIB210 ascites (i.p) to deplete CD4^+^ and CD8^+^ cells. After 48 hr, DN T cells were purified using mouse CD90.2 selection kit (StemCell Technologies, Vancouver, BC). Enriched DN T cells were > 99% negative for CD4 and CD8 expression and > 95% positive for CD3 by flow cytometry.

### Isolation of cells from infected footpads

To assess the numbers (percentages) and proliferation of DN T cells at the site of infection, CFSE-labeled whole T cells from *L. major*-infected Thy1.2 mice were adoptively transferred into naïve Thy1.1 mice that were then challenged with *L. major*. After 7 days, recipient mice were sacrificed and donor cells were recovered from the footpads as we previously described [41]. Briefly, the footpads were disinfected in 70% ethanol, the skins were peeled off and homogenized gently in PBS with tissue grinders. The crude homogenates were resuspended in 7 ml of cold PBS, carefully layered on top of 5 ml Ficoll and the infiltrating cells were separated by centrifugation according to the manufacturer's suggested protocols. The cells were collected, resuspended in 5 ml complete medium, counted, stained directly for expression of various cell surface markers and analyzed by flow cytometer by gating on Thy1.2^+^ donor cells.

### RT-PCR, PCR array and qPCR

For reverse transcription-PCR (RT-PCR), cells from spleens of healed mice were stained with fluorescent-conjugated anti-CD3, anti-CD4 and anti-CD8 antibodies. CD4, CD8 and DN T cells were sorted to high purity by gating on CD3^+^ cells. CD4, CD8 and GAPDH gene expression in sorted cells were analyzed by RT-PCR. For PCR array, CFSE-labeled whole spleen cells from healed mice were stimulated with *L. major*-infected BMDCs for 5 days and proliferating (CFSE^lo^) CD4^+^ and DN T cells were purified by cell sorting. Eighty-four innate and adaptive immune genes in CD4^+^ and DN T cells were analyzed with Mouse Innate & Adaptive Immune Responses PCR Array kit (Qiagen, Frederick, MD). PCR array was performed by a real-time cycler (Bio-Rad CFX96) and analyzed with web-based PCR Array Data Analysis Software (Qiagen, Frederick, MD). To quantify gene expression levels, equal amounts of cDNA were mixed with SYBR Green PCR master mix (Toyobo, Osaka, Japan) and primers specific for the gene of interest (Table S1). 18S rRNA was amplified as an internal control.

### BrdU treatment

Naïve and healed mice were injected with 2 mg of BrdU i.p. per mouse and then challenged with 5×10^6^ *L. major* in the next day. BrdU solution was prepared in sterile water, protected from light exposure, and changed daily. The night before the assay, mice were injected i.p. with 0.8 mg of BrdU in PBS. The next day, mice were sacrificed, spleens were harvested and BrdU staining was performed using BrdU Staining Kit according to the manufacturer's suggested protocol (BD PharMingen).

### Antibody depletion *in vivo*

Healed mice were depleted of CD4 and/or CD8 T cells by injecting i.p. 200 µl ascites containing anti-CD4 (GK1.5) or anti-CD8 (TIB 210) mAb (or both) per mouse or depleted of total T cells by injecting i.p. 100 µg anti-Thy1.2 mAb (TIB 107) per mouse, once a week, and then challenged with 5×10^6^ *L. major*.

### Statistics

Data are presented as means and standard error of mean (SEM). Two-tailed Student's t-test or ANOVA were used to compare means and SEM between groups using GraphPad Prism software. Differences were considered significant at p<0.05.

## Supporting Information

Figure S1 **CD3^+^CD4^−^ (DN) T cells from CD8^+^ T cell-depleted mice proliferate and produce IFN-γ in response to** ***L. major*** **-infected BMDCs stimulation** ***in vitro*** **.** *L. major*-infected mice were depleted *in vivo* of CD8^+^ cells (by i.p. injection of 200 µl TIB210 ascites) 48 hr before sacrifice. Cell depletion in dLNs and spleens was assessed by flow cytometry (A). Purified T (Thy.2^+^) cells from CD8^+^ T cell-depleted mice were stimulated with *L. major*-infected BMDCs for 5 days and cell proliferation and IFN-γ production by CD4^+^ and DN T cells were analyzed by flow cytometry after gating on CD3^+^ cells (B).
(TIF)

Figure S2 **DN T cells proliferate and produce pro-inflammatory cytokines in response to** ***L. major*** **-infected BMDCs** ***in vitro*** **.** Purified CFSE-labeled T cells from spleens of *L. major*-infected and healed C57BL/6 mice (> 12 weeks) were co-cultured with *L. major*-infected BMDCs for 5 days and proliferation and cytokine production were analyzed by flow cytometry following gating on CD3^+^ T cells. Shown are histogram (A) and bar graphs (B-D) showing the frequency of IL-17-producing (A), total proliferating cells (B) and the MFI of proliferating (CFSE^lo^) IFN-γ- (C) and TNF- and (D) producing CD4^+^ and DN T cells *, p<0.05, ***, p<0.001.
(TIF)

Figure S3 **Dot plots showing the sorting strategy and purity of CD4^+^, CD8^+^ and DN T cells for** ***in vitro*** **stability and inhibitory culture experiments.** CD4^+^, CD8^+^ and DN T cells were purified by cell sorting after gating on CD3^+^ (T) and CD19^−^ (B) cells. The sorted cells were used to analyze CD4 and CD8 mRNA transcripts by RT-PCR (Fig. 4B) or cultured *in vitro to* assess stability of CD4 and CD8 molecule expression (Fig. 4C) and suppressive ability of DN T cells on CD4^+^ T cell proliferation and IFN-γ production (Fig. 4D).
(TIF)

Figure S4 ***In vivo*** **cell depletion of CD4^+^ and CD8^+^ T cells to assess the role of DN cells in primary immunity in** **Fig 7A-C** **.** Naïve C57BL/6 mice were injected with anti-CD4 and anti-CD8 mAb, anti-Thy1.2 mAb or rat IgG (control) and then challenged with 5×10^6^ *L. major* 3 days after antibody treatment. Antibody treatment was continued once weekly for 3 weeks when mice were sacrificed. Pooled dLN cells and splenocytes were assessed directly *ex vivo* for CD3, CD4 and CD8 expressions by flow cytometry.
(TIF)

Figure S5 **DN T cells control parasite growth in infected macrophages with** ***L. major*** **.** Purified DN cells from healed or naïve mice (A, B) or CD4^+^ cells from healed mice (B) were co-cultured with *L. major*-infected BMDMs. After 72 hours, cytospin preparations were made, stained with Wright-Giemsa staining and parasite numbers in 100 random cells were determined microscopically. *, p<0.05.
(TIF)

Figure S6 **Purity of DN cells used for adoptive transfer experiment in** **Fig 7E** **.** Healed or naïve mice were injected with ascites fluids containing anti-CD4 and anti-CD8 mAb. After 3 days, DN T cells were purified from splenocytes using CD90 positive selection kit. CD90^+^ cell purity (A) and the expression of CD4 and CD8 molecules (B) on purified cells were assessed by flow cytometry.
(TIF)

Figure S7 **DN T cells home and proliferate at the primary infection site.** CFSE-labeled whole spleen cells from healed CD90.2 mice were adoptively transferred into naïve CD90.1 recipients that were challenged with 5 × 10^6^ *L. major* the next day. Seven days after challenge, mice were sacrificed and donor (CD90.2) cells in the footpads were assessed for CD8 and CD4 expression by flow cytometry. In addition, the proliferation of CD4^+^ and DN cells was also assessed.
(TIF)

Figure S8 **DN T cells require memory CD4^+^ T cells for maximal effector response** ***in vitro*** **and** ***in vivo*** **.** Purified DN T cells (1×10^5^) from healed mice were co-cultured with equal numbers of CD4^+^ T cells from healed or naïve mice in the presence of *L. major*-infected BMDCs. An added control of DN T cells only without CD4^+^ T cells was also included. After 5 days, the proliferation of DN cells was analyzed by flow cytometry (A). Highly enriched (> 98%) CFSE-labeled DN or CD90.2^+^ T cells from healed Thy1.2 mice were adoptively transferred into naïve Thy1.1 recipients that were challenged with 5 × 10^6^ *L. major* the next day. Seven days after challenge, mice were sacrificed and cell proliferation and IFN-γ production by DN T cells were analyzed directly *ex vivo* by gating on Thy1.2^+^CD3^+^CD4^−^CD8^−^ (donor) cell population (B).
(TIF)

Table S1 **Primer sequences used in qRT-PCR to validate differentially regulated genes between CD4^+^ and DN T cells as observed in the PCR array assay.**
(DOCX)
