# Supplementary figures and images for: MHC Class II Restricted Innate-Like Double Negative T Cells Contribute to Optimal Primary and Secondary Immunity to Leishmania major
Source: PLoS Pathog. 2014 Sep 18;10(9):e1004396. doi: 10.1371/journal.ppat.1004396 (PMC4169504; doi:10.1371/journal.ppat.1004396)

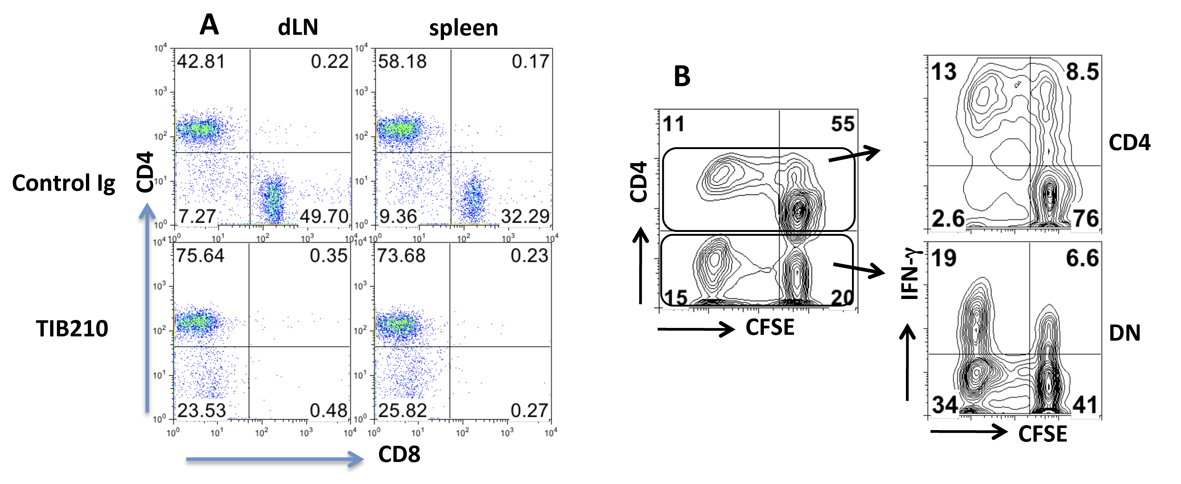

Supplement: Figure S1 — CD3+CD4− (DN) T cells from CD8+ T cell-depleted mice proliferate and produce IFN-γ in response to L. major -infected BMDCs stimulation in vitro . L. major-infected mice were depleted in vivo of CD8+ cells (by i.p. injection of 200 µl TIB210 ascites) 48 hr before sacrifice. Cell depletion in dLNs and spleens was assessed by flow cytometry (A). Purified T (Thy.2+) cells from CD8+ T cell-depleted mice were stimulated with L. major-infected BMDCs for 5 days and cell proliferation and IFN-γ production by CD4+ and DN T cells were analyzed by flow cytometry after gating on CD3+ cells (B). (TIF) [file ppat.1004396.s001.tif]

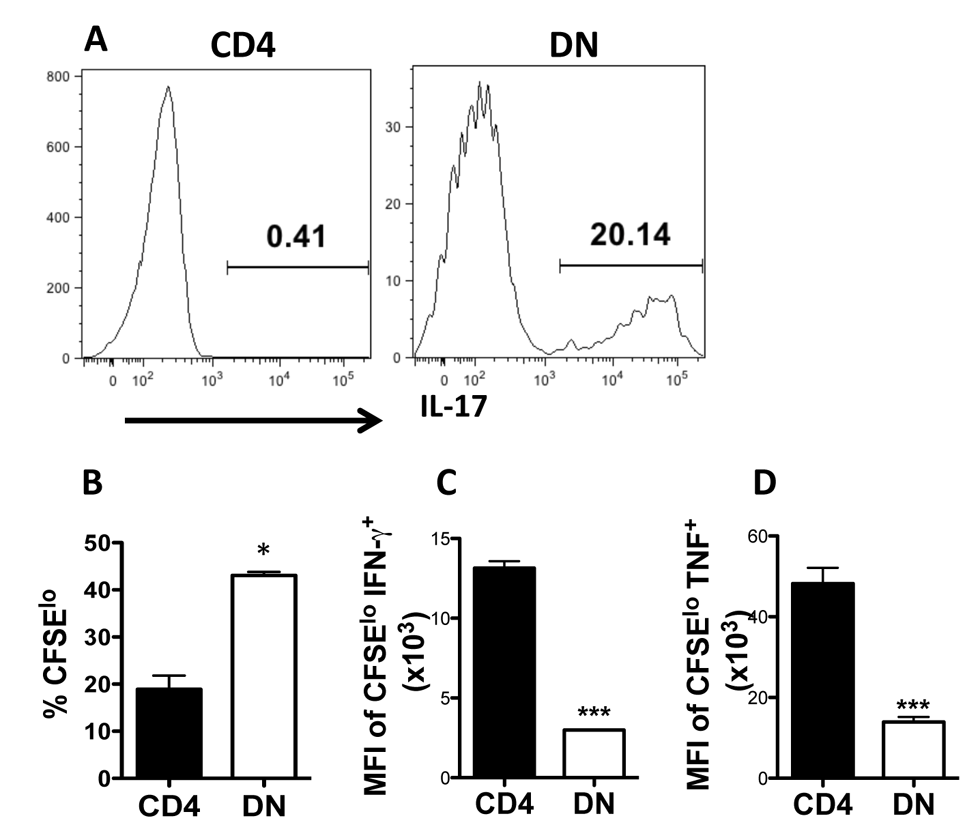

Supplement: Figure S2 — DN T cells proliferate and produce pro-inflammatory cytokines in response to L. major -infected BMDCs in vitro . Purified CFSE-labeled T cells from spleens of L. major-infected and healed C57BL/6 mice (> 12 weeks) were co-cultured with L. major-infected BMDCs for 5 days and proliferation and cytokine production were analyzed by flow cytometry following gating on CD3+ T cells. Shown are histogram (A) and bar graphs (B-D) showing the frequency of IL-17-producing (A), total proliferating cells (B) and the MFI of proliferating (CFSElo) IFN-γ- (C) and TNF- and (D) producing CD4+ and DN T cells *, p<0.05, ***, p<0.001. (TIF) [file ppat.1004396.s002.tif]

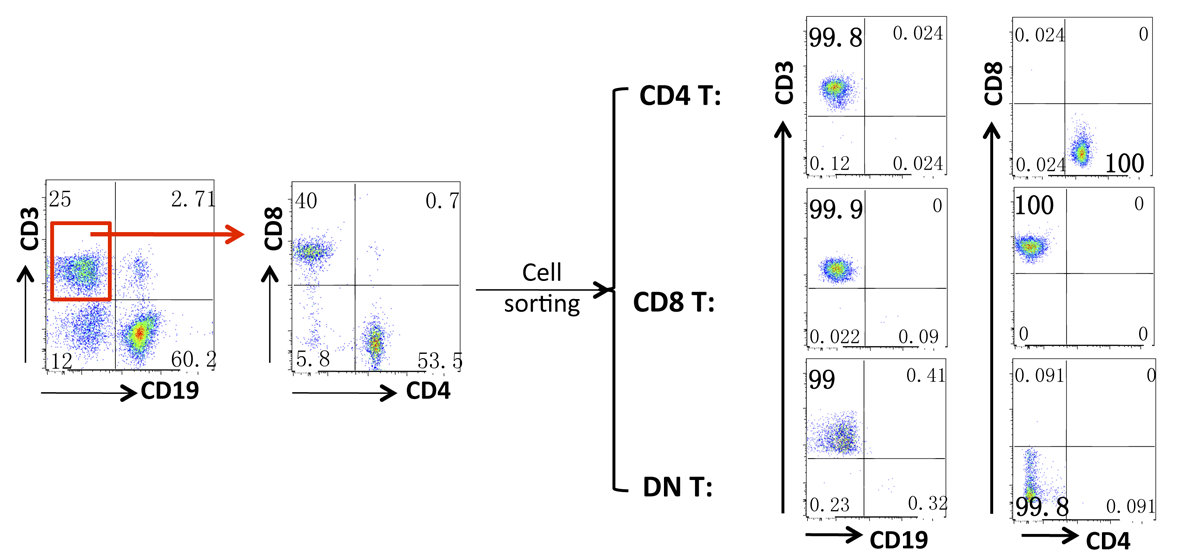

Supplement: Figure S3 — Dot plots showing the sorting strategy and purity of CD4+, CD8+ and DN T cells for in vitro stability and inhibitory culture experiments. CD4+, CD8+ and DN T cells were purified by cell sorting after gating on CD3+ (T) and CD19− (B) cells. The sorted cells were used to analyze CD4 and CD8 mRNA transcripts by RT-PCR (Fig. 4B) or cultured in vitro to assess stability of CD4 and CD8 molecule expression (Fig. 4C) and suppressive ability of DN T cells on CD4+ T cell proliferation and IFN-γ production (Fig. 4D). (TIF) [file ppat.1004396.s003.tif]

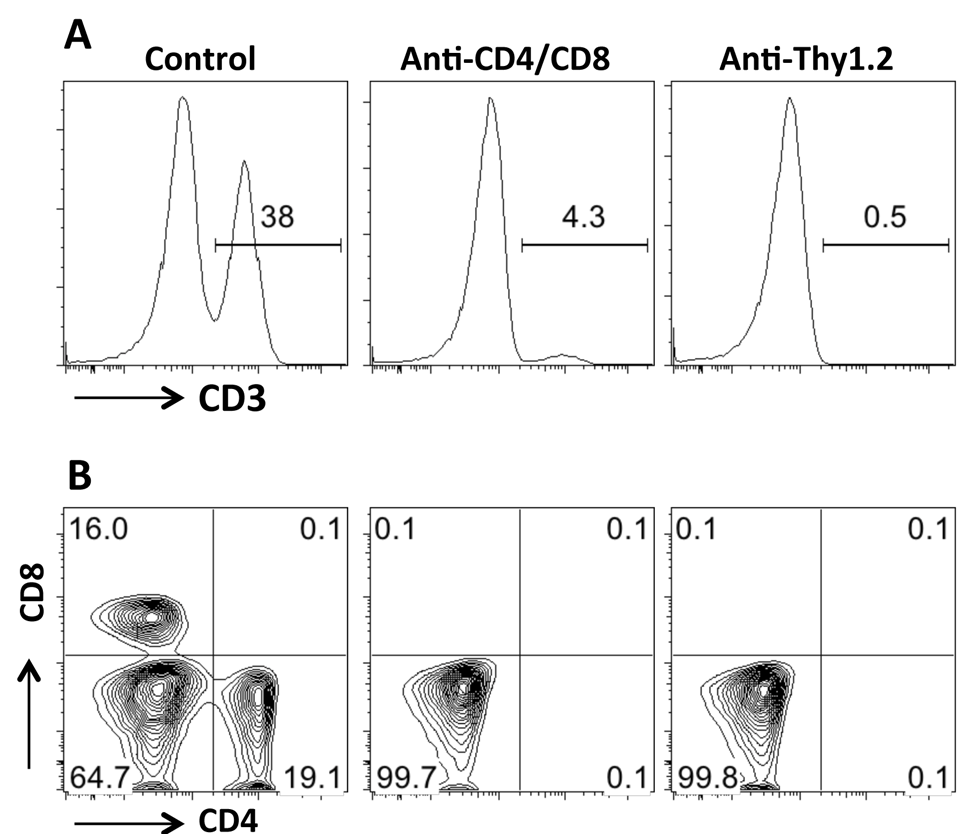

Supplement: Figure S4 — In vivo cell depletion of CD4+ and CD8+ T cells to assess the role of DN cells in primary immunity in Fig 7A-C . Naïve C57BL/6 mice were injected with anti-CD4 and anti-CD8 mAb, anti-Thy1.2 mAb or rat IgG (control) and then challenged with 5×10^6^ L. major 3 days after antibody treatment. Antibody treatment was continued once weekly for 3 weeks when mice were sacrificed. Pooled dLN cells and splenocytes were assessed directly ex vivo for CD3, CD4 and CD8 expressions by flow cytometry. (TIF) [file ppat.1004396.s004.tif]

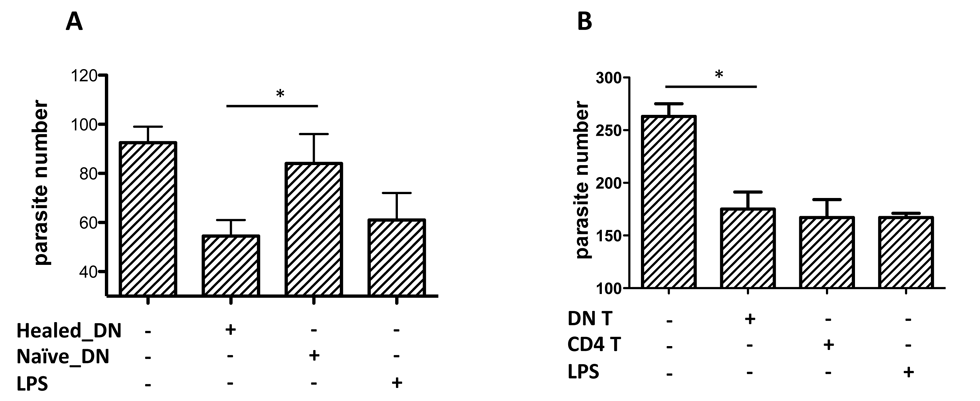

Supplement: Figure S5 — DN T cells control parasite growth in infected macrophages with L. major . Purified DN cells from healed or naïve mice (A, B) or CD4+ cells from healed mice (B) were co-cultured with L. major-infected BMDMs. After 72 hours, cytospin preparations were made, stained with Wright-Giemsa staining and parasite numbers in 100 random cells were determined microscopically. *, p<0.05. (TIF) [file ppat.1004396.s005.tif]

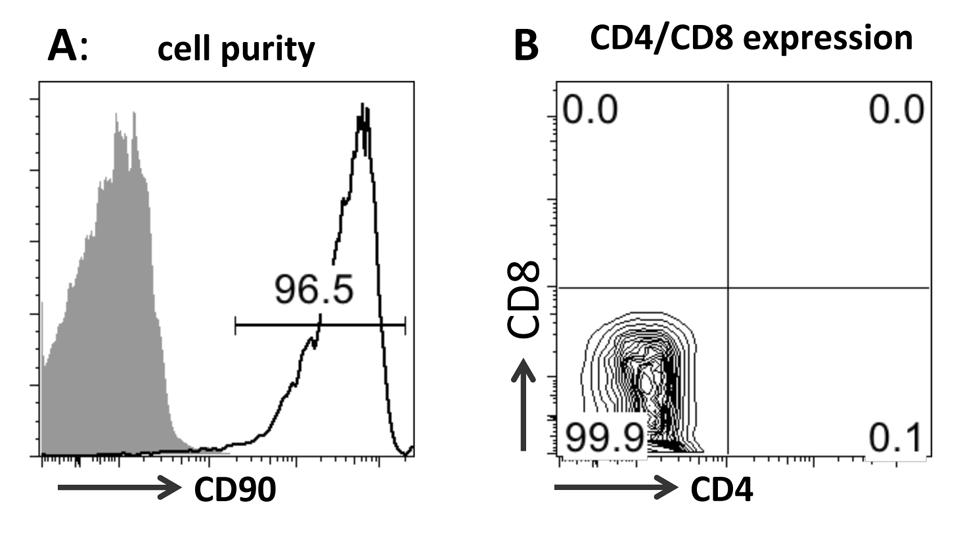

Supplement: Figure S6 — Purity of DN cells used for adoptive transfer experiment in Fig 7E . Healed or naïve mice were injected with ascites fluids containing anti-CD4 and anti-CD8 mAb. After 3 days, DN T cells were purified from splenocytes using CD90 positive selection kit. CD90+ cell purity (A) and the expression of CD4 and CD8 molecules (B) on purified cells were assessed by flow cytometry. (TIF) [file ppat.1004396.s006.tif]

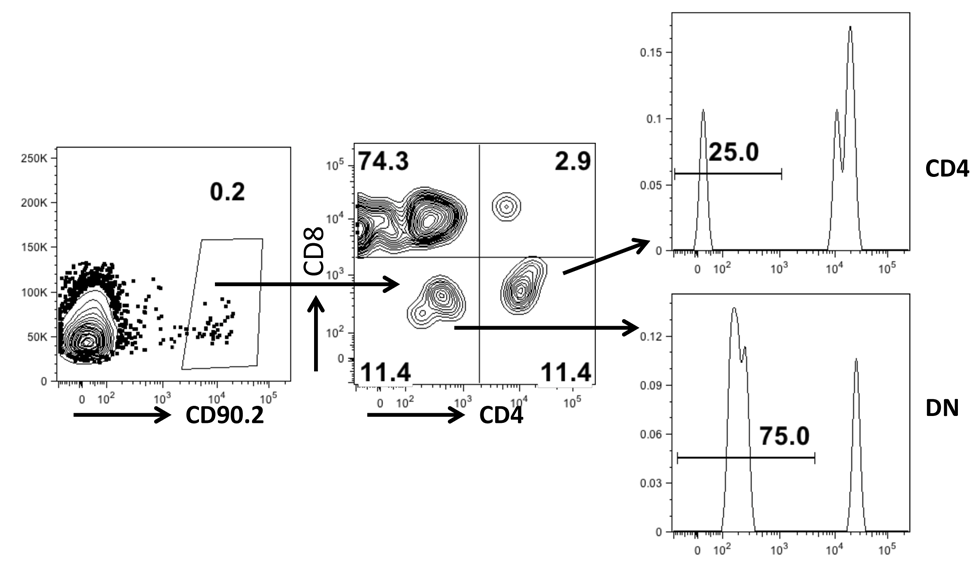

Supplement: Figure S7 — DN T cells home and proliferate at the primary infection site. CFSE-labeled whole spleen cells from healed CD90.2 mice were adoptively transferred into naïve CD90.1 recipients that were challenged with 5 × 10^6^ L. major the next day. Seven days after challenge, mice were sacrificed and donor (CD90.2) cells in the footpads were assessed for CD8 and CD4 expression by flow cytometry. In addition, the proliferation of CD4+ and DN cells was also assessed. (TIF) [file ppat.1004396.s007.tif]

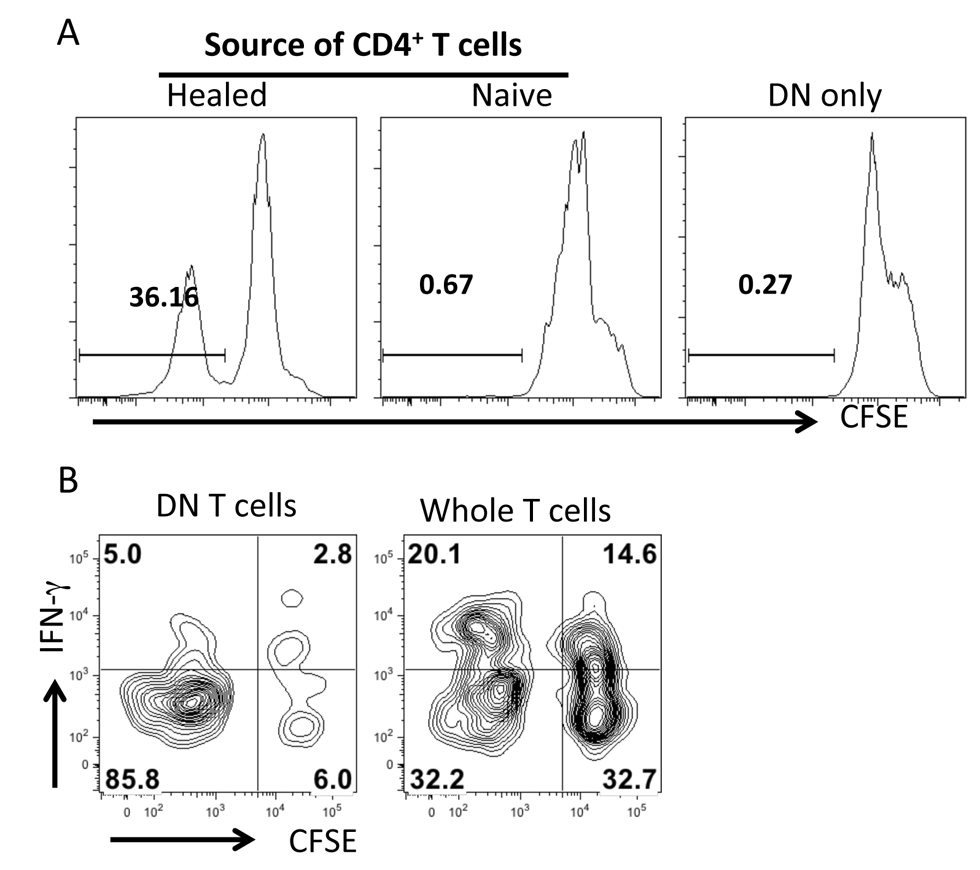

Supplement: Figure S8 — DN T cells require memory CD4+ T cells for maximal effector response in vitro and in vivo . Purified DN T cells (1×10^5^) from healed mice were co-cultured with equal numbers of CD4+ T cells from healed or naïve mice in the presence of L. major-infected BMDCs. An added control of DN T cells only without CD4+ T cells was also included. After 5 days, the proliferation of DN cells was analyzed by flow cytometry (A). Highly enriched (> 98%) CFSE-labeled DN or CD90.2+ T cells from healed Thy1.2 mice were adoptively transferred into naïve Thy1.1 recipients that were challenged with 5 × 10^6^ L. major the next day. Seven days after challenge, mice were sacrificed and cell proliferation and IFN-γ production by DN T cells were analyzed directly ex vivo by gating on Thy1.2+CD3+CD4−CD8− (donor) cell population (B). (TIF) [file ppat.1004396.s008.tif]
