# Supplementary material for: MHC Class II Restricted Innate-Like Double Negative T Cells Contribute to Optimal Primary and Secondary Immunity to Leishmania major
Source: PLoS Pathog. 2014 Sep 18;10(9):e1004396. doi: 10.1371/journal.ppat.1004396 (PMC4169504; doi:10.1371/journal.ppat.1004396)
Supplement: Table S1 — Primer sequences used in qRT-PCR to validate differentially regulated genes between CD4+ and DN T cells as observed in the PCR array assay. (DOCX) [file ppat.1004396.s009.docx]

Table S1

| Mpo F | 5’-CCTCATTGGCACTCAGTTTAG-3’ |
| --- | --- |
| Mpo R | 5’-CCAGTGTTGTCACAGATGATAC-3’ |
| C3 F | 5’-TCAATGAGCAAAGATACTACGG-3’ |
| C3R | 5’-GGACACATCCATGTTCAAGT-3’ |
| Ccr4 F | 5’-TCATTCACTGCTGCCTTAAC-3’ |
| Ccr4 R | 5’-GAGTAGACCTGGAGGAAGTC-3’ |
| Ifng F | 5’-CAAGTGGCATAGATGTGGAA-3’ |
| Ifng R | 5’-CAAAGAGTCTGAGGTAGAAAGAG-3’ |
| 18s F | 5’-TGA CTC AAC ACG GGA AAC CTC A-3’ |
| 18s R | 5’-ACC AGA CAA ATC GCT CCA CCA A-3’ |
